# Agent-Based Modeling of Endotoxin-Induced Acute Inflammatory Response in Human Blood Leukocytes

**DOI:** 10.1371/journal.pone.0009249

**Published:** 2010-02-18

**Authors:** Xu Dong, Panagiota T. Foteinou, Steven E. Calvano, Stephen F. Lowry, Ioannis P. Androulakis

**Affiliations:** 1 Department of Biomedical Engineering, Rutgers University, Piscataway, New Jersey, United States of America; 2 Department of Surgery, University of Medicine and Dentristry of New Jersey Robert Wood Johnson Medical School, New Brunswick, New Jersey, United States of America; Fondazione Telethon, Italy

## Abstract

**Background:**

Inflammation is a highly complex biological response evoked by many stimuli. A persistent challenge in modeling this dynamic process has been the (nonlinear) nature of the response that precludes the single-variable assumption. Systems-based approaches offer a promising possibility for understanding inflammation in its homeostatic context. In order to study the underlying complexity of the acute inflammatory response, an agent-based framework is developed that models the emerging host response as the outcome of orchestrated interactions associated with intricate signaling cascades and intercellular immune system interactions.

**Methodology/Principal Findings:**

An agent-based modeling (ABM) framework is proposed to study the nonlinear dynamics of acute human inflammation. The model is implemented using NetLogo software. Interacting agents involve either inflammation-specific molecules or cells essential for the propagation of the inflammatory reaction across the system. Spatial orientation of molecule interactions involved in signaling cascades coupled with the cellular heterogeneity are further taken into account. The proposed in silico model is evaluated through its ability to successfully reproduce a self-limited inflammatory response as well as a series of scenarios indicative of the nonlinear dynamics of the response. Such scenarios involve either a persistent (non)infectious response or innate immune tolerance and potentiation effects followed by perturbations in intracellular signaling molecules and cascades.

**Conclusions/Significance:**

The ABM framework developed in this study provides insight on the stochastic interactions of the mediators involved in the propagation of endotoxin signaling at the cellular response level. The simulation results are in accordance with our prior research effort associated with the development of deterministic human inflammation models that include transcriptional dynamics, signaling, and physiological components. The hypothetical scenarios explored in this study would potentially improve our understanding of how manipulating the behavior of the molecular species could manifest into emergent behavior of the overall system.

## Introduction

The acute inflammatory response (AIR) is the initial response of the host to a diverse array of biological stressors including infection, burns, trauma and invasive surgery. Under normal circumstances the dynamics of acute inflammation are tightly regulated and self-limited [1]; however when anti-inflammatory processes fail an amplified inflammatory state is characterized by severe, uncontrolled systemic inflammation and multiple organ dysfunction can develop [2].

Despite the growing understanding of the cellular and molecular mechanisms of systemic inflammation [3] the complexity of the response has challenged therapeutic development [4], [5]. A key reason for this conundrum has been speculated to be the difficulty of predicting the impact of manipulating individual components of the highly complex, non-linear and redundant inflammatory response [6]. Thus progress would require a greater understanding of how components are organized to generate a behavior thus making systems based approaches appealing. Mathematical modeling as a dynamic knowledge representation offers a promising possibility for understanding complex physiologic responses in their homeostatic context. As a result, various approaches have been proposed to simulate the underlying complexity of the inflammatory response including both equation based models (EBM) and agent based models (ABM) [7], [8].

Although both modeling approaches (EBM and ABM) have both advantages and disadvantages [8], agent based modeling has emerged as an alternative for addressing features of complex biological systems [9]. The recognition that EBM are predicated on the assumption of a homogeneously distributed system has made it less applicable in situations where spatial effects are important [10]. On the other hand, ABM has an intrinsically spatial component based on its reliance upon local interactions and environmental heterogeneity. To examine the effects of the assumption of spatial heterogeneity, there is a growing body of research probing the effects of spatial distribution in the innate immune system [11], [12]. Biological systems, unlike physical or chemical systems are characterized by the emergence of inhomogeneous distribution of their components [13]. Thus, a central premise of ABM is that they map intuitively to biological phenomena such as cells within tissues and organs capturing the stochastic nature and dynamic transitional states in biological systems [14], [15], [16], [17]. In addition to this, the ABM approach provides a very intuitive means of translation of basic science data on the innate immune response through a series of simple rules that dictate their behaviors. Accordingly, a number of excellent prior studies have placed significant emphasis on simulating the dynamics of inflammation predicated upon the principles of agent based models [18], [19], [20], [21], [22]. Specifically, in the studies conducted by An and collaborators [10], [23], [24], [25], the applications of ABM in inflammation models have been effectively demonstrated. Their work showed the considerable potential of agent-based modeling of biological systems and has motivated the design of the model in this paper.

The key elements in ABMs are the agents, which are entities that represent a certain aspect of the system, for instance a family of cells and/or molecules that are able to adapt and interact with the environment and with each other based on a specific set of rules [26]. While agents within a class will have the same rules for behavior, the behavior of individual agents varies because of differences in local conditions. The individual interactions then aggregate to engender the overall behavior observed in an experimental setting. The advantage of ABMs lies in the fact that the interactions of agents are derived from fundamental occurrences in biological processes, like the binding of molecules, and as such, they are more intuitive to implement and easier to understand. Additionally, the instructions that describe the interactions are taken from published literature and translated into programming language. Furthermore, the model is naturally stochastic in that the interactions can be designed to be based upon probabilities and some of the agent dynamics can be highly random.

The work discussed in this report seeks to address the possibility of an agent based modeling approach that defines the propagation of a perturbation across the system taking into account spatial orientation at the molecular level as well as cellular interactions and heterogeneity. Driven by the premise that peripheral blood leukocytes (PBLs) are major effectors in response to endotoxin and that PBLs represent a composite mixture of several cellular subpopulations we opted to simulate the stochastic interactions particularly in the macrophages and T helper cells. During the onset of the inflammatory response, the secretion of pro-inflammatory cytokines from macrophages stimulates the activation of precursor T helper cells (Th0) and induce them to exhibit the type 1 T helper cell (Th1) phenotype thatin turn facilitates the secretion of various pro-inflammatory cytokines [27]. The other fate of Th0 is to become type 2 T helper cells (Th2) and produce anti-inflammatory cytokines that are essential for restoring homeostasis [28]. Physiologically, the recruitment of macrophages and the differentiation of Th cells occur in separate locations. Yet they retain strong interconnectivity facilitated by the inflammatory cytokines. Due to limitation of the framework, the proposed model did not separate the aforementioned cell types into different topological compartments. We assumed however that the movement of the cytokine agents from the macrophages to the Th cells would signify the transportation of the cytokines between different biological tissues. Previous agent based studies have placed emphasis on simulating either intercellular interactions between a multitude of such cell types [29] or the spatial orientation of molecules involved in the NF-kB signaling pathway [30] while considerable attention has been also given to modeling the transcriptional regulatory network of TH differentiation [31]. In this paper we have taken an integrative approach to elucidate molecular interactions involved in the NF-kB signaling pathway, coupled with the spatial orientation of various inflammation specific molecules and cell populations such as macrophages and T-helper cells. At the transcriptional response level, we have previously demonstrated that the transcriptional dynamics of human leukocytes exposed to bacterial endotoxin can be decomposed into to three elementary comprehensive responses [32], [33]. These responses defined the major (essential) transcriptional elements of the host response to endotoxin that subsequently manifest the integrated systemic response. In an attempt to establish quantifiable relationships among these essential components of human endotoxemia we have proposed the development of deterministic, semi-mechanistic based host response models that include transcriptional dynamics, signaling and physiological components for the modulation of the response [33], [34].

Our agents of choice reflected the characteristics of biological molecules. This allowed us to focus on the intracellular dynamics of the NF-kB signaling module and further illustrate the subsequent intercellular interactions through the up-regulation of inflammatory mediators. The stochastic behavior of the agents was partially attributed to the random motion of the molecules. The probability that determined whether an interaction should occur relied on the spatial configuration of the participants. Cells were not considered as reactor spaces with an even distribution of molecules. To accommodate for this, some of the agent-based rules regarding the mobilization of molecules were implemented (**Table 1**), in order to ensure that a specific interaction occurs within an allocated time frame and the network topology of the model. A key characteristic of our approach was to represent the cellular interactions as the aggregated output of an intricate process that influenced the cellular behavior and therefore the overall systemic response.

**Table 1:** List of agent based rules.

Agents	Agent rules
Macrophages	Produce 1 unit of IKK every 5 ticks
	Move towards the direction that has the highest LPS count within 5 + cell radius
	Bind free LPS molecules with unoccupied receptors
IKK	Activated by the formed TNF-TNFR complex
	70% chance to bind to inactive NF-kB
	Dissociate from the complex after 10 ticks
	Stimulates Nf- kB, IkBa is ubiquitinated
	Deactivated by activated IkBa as a result of the transcriptional activity of NF-kB
	Degrades after a random of 1 to 799 ticks
NF-kB	If activated and translocates to the nucleus, asks the macrophage to produce 1 IkBa every 10 ticks, 1 unit of IL-12 with 80% chance and 1 unit of TNF-a
IkBa	If activated, seek out activated NF-kB within radius of 1
	Bind to any activated NF-kB, form a complex and both members of the complex become inactivated
Receptors	TLR4 (LPS receptors) become activated when bind to LPS molecules. If activated, then produce 1 unit of TNF-a every 100 clicks
	IL-4 and IL-12 receptors receive their respective targets (receptors)
IL-4	In the presence of free IL-12, 3 units of IL-4 are produced by 86% for every 1 to 5 clicks
	Bind to IL-4 receptors on Th-0 cells or macrophages. On macrophages, the binding rate increases the energetic level of the macrophage by 1
IL-12	Produced by macrophages and Th-1 cells
	Bind to IL-12 receptors on Th-0 directing the differentiation towards either Th-1 orTh-2
Th cells	Th-0 cells count the unit of interleukins on its surface receptors. Once the number of interleukins reaches 25, then differentiate into either Th-1 or Th-2. If more IL-12 molecules are present than IL-4, then become Th-1. Otherwise, it becomes a Th-2 type
LPS	Frequency of movement: 800 times more frequent than other cellular agents
	Collides with LPS receptors on the surface of macrophages
	Activates the receptor while the “sensitivity value” decreases by 1
	A successful binding occurs if a random value between 1 and the maximum value of sensitivity (which equals to 5) is less than the current sensitivity
	Activated endotoxin receptors produce 1 unit of TNF-a
	If binds to its receptor, then degrade 1 to 2 ticks
TNF-a	Start with 600 ticks
	Binds to TNFR on the surface of macrophages
	Amplifies intracellular IKK activity
	If it binds to a receptor, then degrade in 50 clicks or in 200 clicks if there is nearby bound IL-4. Degrades by 1 click naturally
Movement restrictions	Intracellular molecules e.g. NF-kB, IkBa, IKK are confined inside the agents of the plasma membrane. Only activated NF-kB and IkBa can enter the nucleus. Before these agents perform any rule based movement, a check is made whether they are facing the membrane, other molecules or the nucleus. If the destination is inaccessible, then they will face another direction until the next move is achieved. Their positions are further updated as the macrophage moves
	Extracellular molecules cannot enter the nucleus have similar restrictions with the plasma membrane agents

Inevitably there is a level of abstraction that needs to be considered when representing molecular reactions as discrete events that follow somewhat arbitrary rules. The validity of our approach will be demonstrated through its potential to reproduce biologically relevant scenarios indicative of the non–linear dynamics of systemic inflammation described as the following scenarios: (1) a self-limited response where the inflammatory stimulus was cleared successfully, (2) a persistent infectious response where the inflammatory stimulus was not eliminated, leading to an aberrant inflammatory response, (3) a persistent non-infectious inflammatory response that can be elicited under high concentrations of the inflammatory stimulus, causing an inflammatory insult that can disturb the dynamics of the host response leading to an unconstrained inflammatory response; and finally, (4) two scenarios associated with endotoxin tolerance and potentiation effects followed by perturbations in the regulatory (NF-kB) signaling module.

## Results and Discussion

### Elements of the Agent Based Host Response Model of Human Inflammation

**Figure 1:**
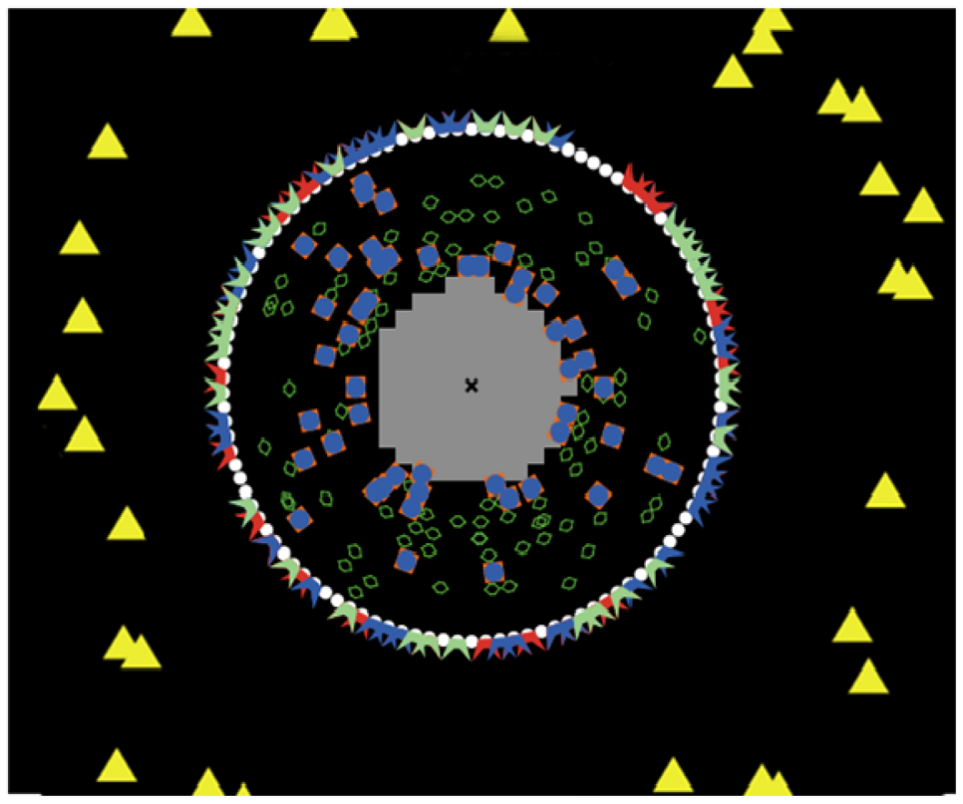
Interacting components/agents involved in the propagation of LPS signaling on macrophages. Yellow triangles reflect the extracellular signal (LPS) and white circles represent the plasma membrane. Red polygons refer to the endotoxin (LPS) receptor and blue polygons refer to the TNF-a receptor. Light green polygons correspond to IL-4 receptors and dark green polygons reflect the presence of kinase (IKK). Blue+orange squares represent the inactive (bound) NF-kB with its inhibitor, IkBa while the grey area refers to the nucleus.

In an effort to study the non-linear dynamics of an *in vivo* human response to endotoxin, we recently proposed a receptor mediated indirect response model that couples extracellular signals with the transcriptional response [32], [33]. The proposed model established quantifiable relationships among the essential components of human inflammation that included the pro-inflammatory response (P) consisting of the early increased expression of cytokines and chemokines; the anti-inflammatory response (A) that served as the immunoregulatory arm of the host defense system; and the energetic response (E) that involved the expression of genes that participate in cellular bioenergetic processes. Driven by the premise that intracellular signaling cascades activated inflammation-specific transcriptional responses, a NF-kB dependent physicochemical host response model was further proposed in [34]. Such a model captured biological information in the form of kinetic rules and signaling cascades for the onset, resolution and control of the inflammatory process. Further, the immune response could be triggered by the activation of the NF-kB signaling module resulting from an activating signal associated with the binding of extracellular signals (LPS) to appropriate receptors (TLR4) [35]. In this study we have sought to describe the host response to endotoxin via interacting molecules and cells based on an integrated ABM framework as shown in **Figure 1**. Accordingly, each macrophage possessed a cell membrane comprised of agents arranged in a circle around the center of the cell which constitutes the nucleus. Receptors for LPS, IL-4, and TNF-alpha were embedded in the membrane while the inhibitor protein IkBa, IKK, and NF-kB are located in the cytosol.

**Figure 2:**
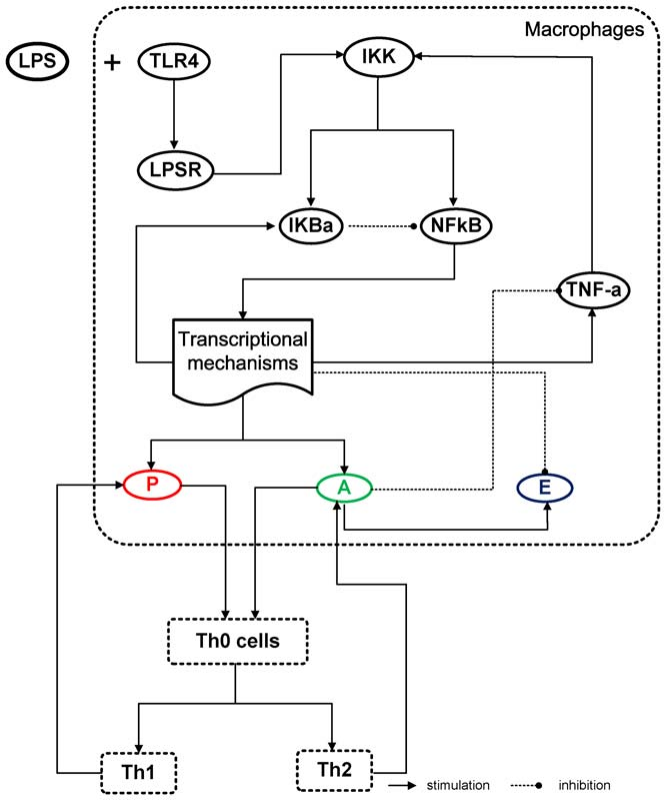
Schematic illustration of elements and interactions involved in the agent based model of endotoxin induced inflammation.

Prior to any external perturbation, NF-kB is inactive in the cytoplasm forming a complex with its primary inhibitor, IkBa. Upon stimulation, NF-kB translocates to the nucleus activating the transcriptional machinery for the up-regulation of the critical transcriptional events [27]–[29]. During the recognition process of LPS from its signaling receptor (R), a signal transduction cascade is triggered that up-regulates the transcription of TNF-a. Since pro-inflammatory cytokines might be responsible for perpetuating and amplifying the inflammatory reaction through the critical node (IKK) [36], such interaction is simulated via the positive interaction between TNF-a, and the kinase activity (IKK). Consequently, the presence of pro-inflammatory mediators (P) promoted the migration of mature T helper cells [37] where Th0 cells become Th1, while the production of anti-inflammatory mediators (A) incited formation of Th2 cells which further potentiate the anti-inflammatory response (A) [28]. Since the anti-inflammatory arm of the host defense system restores homeostasis, the anti-inflammatory component of the model, including anti-inflammatory mediator agents (A), was assumed to exert its counter-regulatory properties by stimulating the degradation rate of the early potent pro-inflammatory mediator TNF-a, coupled with the active populations of T helper cells. In particular, the Th2 agents continuously produced anti-inflammatory mediators to ensure that the population of (A) agents was sufficient to attenuate TNF-a production in macrophages. Since circulating pro-inflammatory (P) agents have the ability to turn Th0 into Th1, instead of Th2, the population of Th2 cells is primarily affected by the concentration of (P) agents. Therefore, the resolution of the inflammatory response is highly dependent on the balance between pro- and anti-inflammatory mediators that are additionally regulated by the energetic state of macrophages. To establish the link between the inflammatory response and the cellular energetic state, we assumed that upon activation of a pre-defined threshold the essential energetic response was assumed to subsequently modulate the degradation rate of TNF-a [38]. The production rate of the inflammatory mediator TNF-a increased when the energetic state was lowered during the progression of the inflammatory reaction by NF-kB. Meanwhile, the presence of anti-inflammatory mediators leads to a decrease in the proximal inflammatory mediator, TNF-a. All the interacting components that constitute the agent based model of inflammation were shown in **Figure 2**.

### Qualitative Assessment of the Model

**Figure 3:**
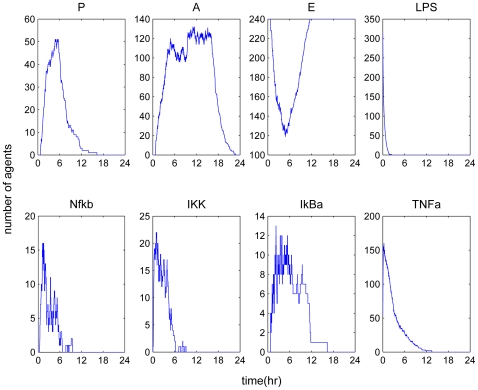
A self-limited inflammatory response (LPS(0) = 350 units). Temporal profiles of essential components that constitute the agent based model resolved within 24 hr.

A self-limited inflammatory response to the endotoxin stimulus corresponds to resolved dynamic profiles for all the elements constituting the model. The objective was to produce the dynamic profiles of a successful inflammatory resolution as shown in **Figure 3** that qualitatively agreed with the previously models using a deterministic approach [33], [34]. While the inflammatory stimulus, namely LPS agents were successfully cleared within 1 h, the activation of anti-inflammatory cytokines expedited the attenuation of the early pro-inflammatory cytokine TNF-a with subsequent termination of the pro-inflammatory signaling cascade. The correctness of the model was evaluated based on its ability to qualitatively predict the uncontrolled responses as below.

#### Implications of increased insult

**Figure 4:**
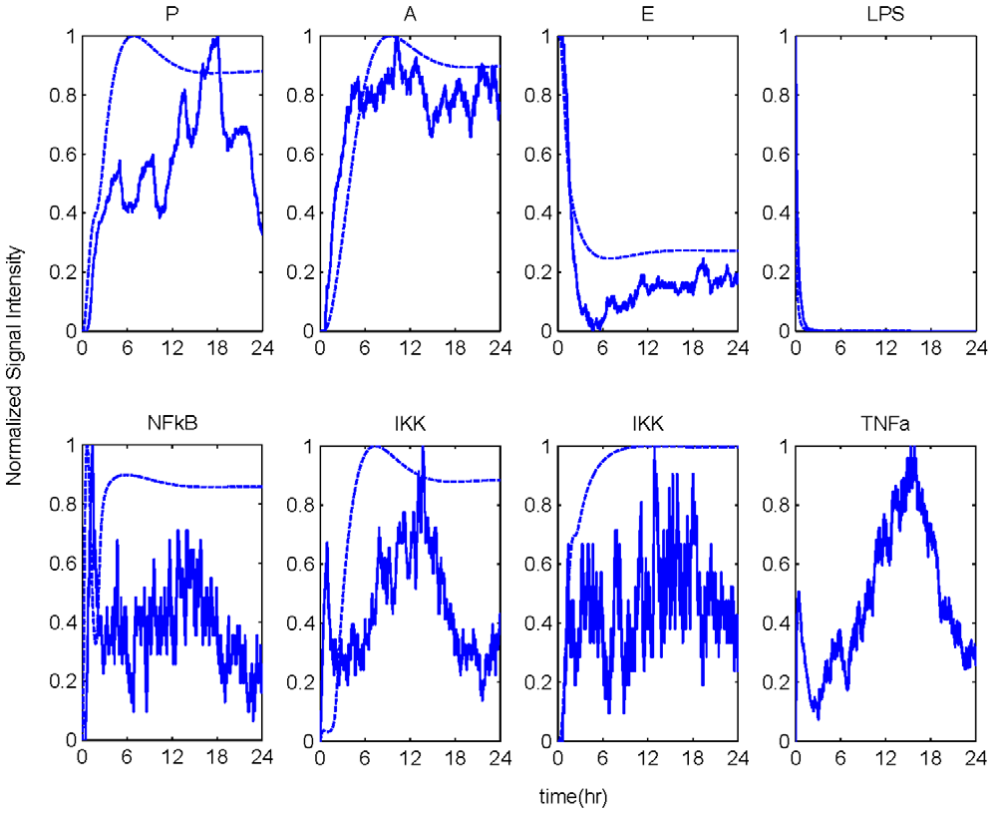
Temporal responses of an unresolved inflammatory response due to high LPS concentration. A high concentration of LPS (LPS(0) = 750) can cause a malfunction in the dynamics of the host leading to an exacerbated inflammatory response (solid lines). Dashed lines refer to the implications of high concentration of LPS as simulated by our deterministic (ODE) approach. For the purpose of comparing the simulated output between the ABM and the ODE model, all responses are normalized so that numerically they range between (0,1).

High concentrations of LPS, corresponding to an increase in the strength of the inflammatory insult, can be responsible for the amplification of the host immune response [39]. This event is followed by a dysregulation in host defense intrinsic dynamics leading to an unconstrained inflammatory response even after the circulating levels of LPS have been cleared. The model predicted the situation where the initial levels of LPS are increased in **Figure 4**. We observed that when the concentration of the inflammatory stimulus exceeded a critical threshold, the inflammatory response did not abate. Such a response is characterized by overwhelming production of the pro-inflammatory instigator, TNF-a, which amplifies the activity of NF-kB. In particular, high LPS concentration potentiates the secretion of pro-inflammatory mediators (P) which in turn may increase the probability of Th0 cells to differentiate into Th1 cells rather than into Th2 cells. Additionally Th1 cells further increase (P) population; thus disturbing the balance between Th1/Th2 accounted for the progression of an unconstrained inflammatory response.

#### Malfunction in LPS clearance rate

**Figure 5:**
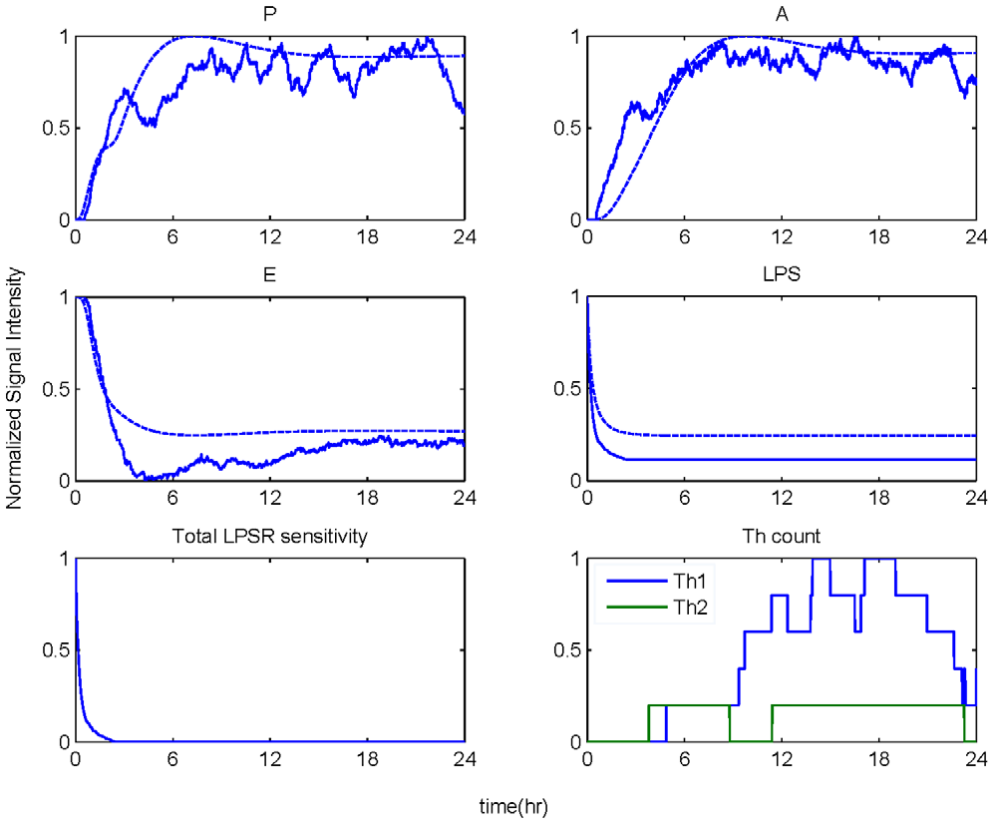
Temporal responses in a persistent infectious inflammatory response. Solid lines correspond to LPS(t = 0 hr) = 1000 which accounts for a prolonged inflammatory activity causing a malfunction in LPS clearance rate. Dashed lines refer to equation-based model predictions for the case of a persistent infectious challenge which can be achieved by manipulating the first order degradation rate of LPS as discussed in the original analysis [34]. The output of both modeling approaches is normalized so that numerically it ranges between (0,1).

An acute pro-inflammatory cytokine “burst” results from intravenous administration of high concentration of LPS into the system of healthy subjects. The subsequent effect is associated with the host's inability to resolve the inflammatory reaction followed by the persistent infectious challenge (unsuccessful clearance of endotoxin) [40], [41]. Accordingly, the prolonged exposure of the system to bacterial infection leads to a significant down-regulation of the endotoxin signaling receptor which further accounts for a slower decay rate causing a dysregulation in the phagocytic capabilities of macrophages [42]. The relevant agent rule that captured such scenario was the “sensitivity” parameter of the endotoxin signaling receptors. As these receptors become saturated during the presence of high amounts of endotoxin, the sensitivity parameter decreases which thereby influences the probability of LPS receptors to be occupied with LPS molecules. During an overwhelming endotoxin challenge, the LPS receptors eventually lose their capability to form additional complexes with LPS, and therefore the LPS agents remain in the system. Although decreased degradation of LPS is not associated with a distinct, well-defined, clinical condition, it is possible that this phenomenon may exist. It is known that triglyceride-rich lipoproteins bind to LPS and that these complexes are cleared by binding to lipoprotein receptors [43]. Furthermore, these receptors are abundant in the liver where ∼70% of lipoproteins are cleared from the circulation. Such malfunction in LPS clearance rate was simulated in **Figure 5**. Similar to the progression of the increased insult scenario as shown in **Figure 4**, the progression of a persistent infectious response was simulated due to the activation of the feedforward loop regarding the activation of IKK which drives downstream an aberrant transcriptional activity of NF-kB and thereby affecting the transcriptional rate of the critical pro-inflammatory mediators, e.g. TNF-a. The secretion of TNF-a further amplified the activity of NF-kB through the critical IKK node [44]. These interactions perturb the dynamics associated with the energetic state of the system. Furthermore, we speculated that a switch-like rule related to the energetic state of the cell can be responsible for the disturbance of the homeostatic production of anti-inflammatory mediators. Such rule has been implemented in the ABM framework in that when the energetic state is below 25% of its original value the production of the anti-inflammatory mediators should increase.

#### Endotoxin hypo-responsiveness

**Figure 6:**
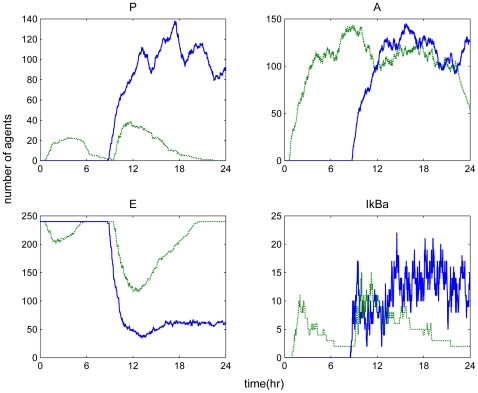
Endotoxin tolerance scenario. Pre-existing infection might cause a profound reduction in cell's capacity (hypo-responsiveness) to respond in the main endotoxin challenge. Solid line: LPS(t = 0 hr) = 750. Dotted line: LPS(t = 0 hr) = 100 & LPS(t = 8 hr) = 650.

The pre-exposure of the host to controlled levels of inflammatory agents affects the eventual fate of the response. It has been observed that repeated doses of endotoxin insult might lead to a less vigorous innate immune response [45]. Such an effect can reverse the lethal outcome of a high dose of the inflammatory stimulus. That is to say, in spite of the potent efficacy of LPS, if the system is pre-exposed to lower sub–lethal doses of LPS then this induces an acquired state of resistance to a subsequent endotoxin challenge [46]. This phenomenon, known as endotoxin hypo-responsiveness is a multifactorial problem that can be associated with decreased TLR signaling by proteins that negatively regulate LPS-induced inflammatory responses [47]. From a modeling standpoint, small dose of LPS is administered 8 hours prior to the main endotoxin insult. Such perturbation modulates the dynamic profiles of both pro-inflammatory and anti-inflammatory mediators as well as the energetic state of the macrophage populations towards resolution within 24 hours. The endotoxin hyporesponsiveness was simulated in **Figure 6** where pre-existing infection caused a profound reduction in cells' capacity to respond to the main (high) endotoxin challenge. There were no agent rules that specified the time interval between the injections that would yield the emergent attenuated response. From a biological standpoint the prior inflammatory insult desensitizes the endotoxin signaling receptors in a manner that these receptors become less sensitive to the subsequent infectious challenge and therefore the cells have enough time to mitigate the endotoxin challenge and resolve the inflammatory reaction.

#### “Lethal” potentiation

**Figure 7:**
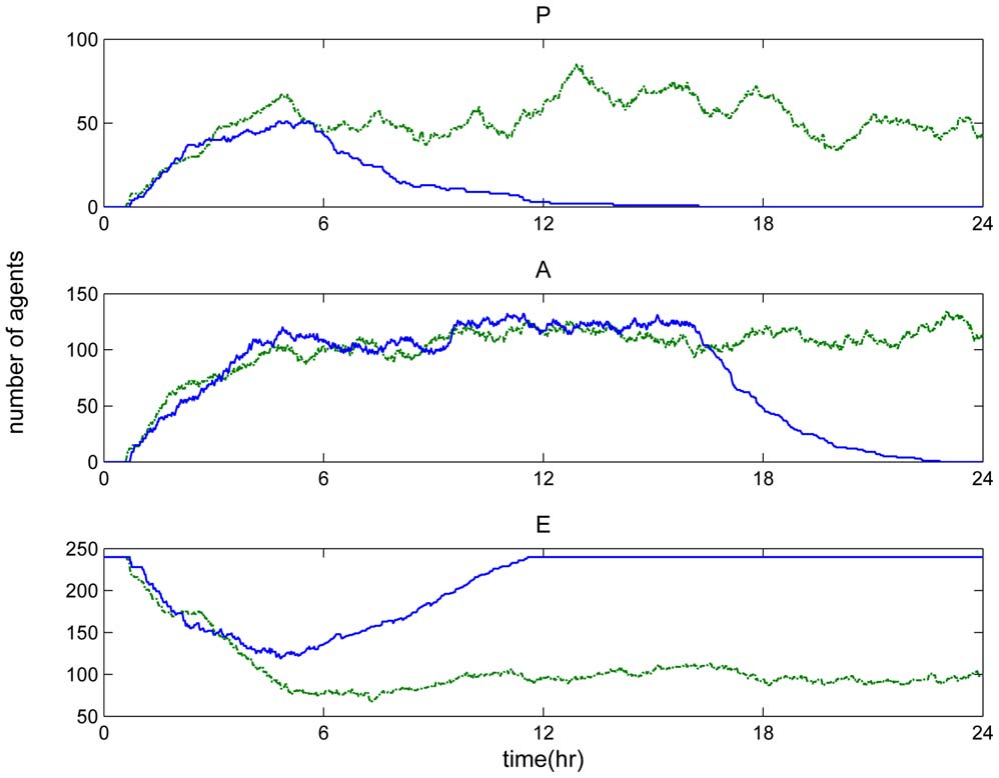
Lethal potentiation. Successive administration of small doses of endotoxin can lead to an unresolved inflammatory response. Solid line: LPS(t = 0 hr) = 350. Dotted line: LPS(t = 0 hr) = 100 & LPS(t = 2 hr) = 250.

Endotoxin hypo-responsiveness is associated with an emergent acquired dynamic state of the system that modulates the response of the system not to respond rigorously to the primary endotoxin challenge. On the other hand, the successive administration of sublethal doses of endotoxin can potentiate the system in that, because of the lack of an acquired state in the dynamics of the system, such an insult may dysregulate the host response dynamics leading to an exacerbated inflammation that cannot resolve. Thus, based on our agent-based model we further explore the behavior of the system when it is either pre-exposed to lower levels of endotoxin for “adequate” time as well as when the system has not manifested its “dynamic memory” to tolerate the second endotoxin challenge [48]. In particular, we simulate such a case administering at t = 0 hr low dose of endotoxin which is shortly followed within 2 hr by another “sub-lethal” insult. From a modeling standpoint, this short time interval was characterized by the accumulation of both pro-inflammatory (P) and anti-inflammatory (A) mediators. The response was exaggerated under conditions of the second endotoxin stimulation due to the priming of various inflammation-specific intracellular signaling molecules which further propagated the inflammatory reaction to nearby cells/agents. The effect of this lethal potentiation scenario was demonstrated in **Figure 7**. Additionally, if the second dosage was administered when the inflammatory mediators are diminishing, then the effect is less prominent due to both the lack of cytokines and the receptor desensitization which occurs due to pre-existing infection.

#### Modulation in the dynamics of NF-kB signaling module

**Figure 8:**
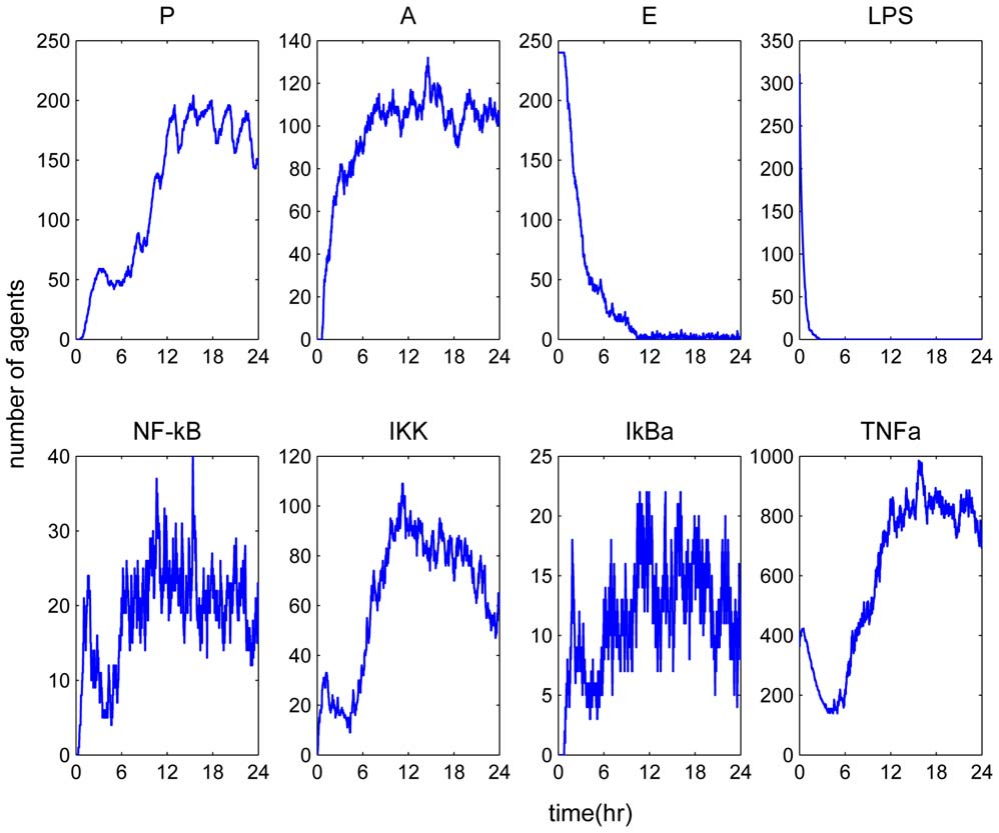
Pre-existence of inflammatory mediators (TNF-a) may enhance abnormally the intracellular signaling amplifying IKK activity. Such response leads to an aberrant inflammatory response which cannot be counter-regulated by the anti-inflammatory arms of the system. Such a mode of dysregulation is simulated by concomitant exposure of the system to TNF-a and bacterial infection (LPS): LPS(t = 0 hr) = 350 & TNF-a(t = 0 hr) = 300.

Another mode of perturbation of the underlying dynamics of the probed system was related to the presence of a “prior” insult that coupled with the LPS stimulus. It accounted for an increased production of pro-inflammatory mediators as shown in **Figure 8**. Such a sustained pro-inflammatory signaling was possible to deregulate the NF-kB signaling module and led to a persistent NF-kB activity [49]. The elevated NF-kB activity implied that the nuclear concentration of NF-kB cannot be further constrained by its primary inhibitor, IkBa and eventually settled to a steady state far away from their equilibrium (homeostasis). We simulated this scenario by pre-conditioning the system with low-dose of TNF-a. Since TNF-a is a potent inflammatory instigator that stimulates IKK activity it can perturb the behavior of the system towards an unbalanced immune response. Clinically, such an increased rate in the production of pro-inflammatory mediators might be the outcome of a surgical trauma followed by bacterial infection, a so called two hit scenario [50].

**Figure 9:**
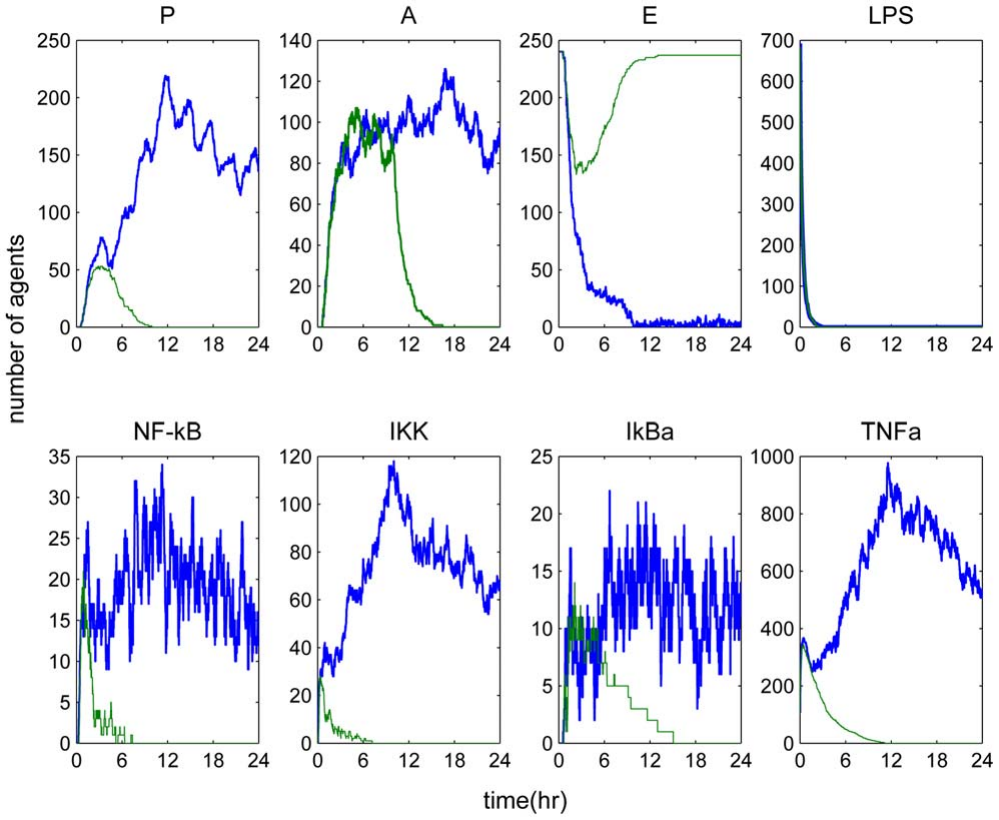
Exploring the effect of an intervention (anti-inflammatory) strategy that inhibits IKK activity. Such scenario is simulated by administering LPS(t = 0 hr) = 750 and IKK inhibitors, IKK inhibitors (t = 0 hr) = 400 (green line).

We have demonstrated the ability of our model to simulate the trajectory of an unconstrained inflammatory response. Further, the potential of the proposed model was also demonstrated through its capability to respond to an intervention strategy that intended to modulate the dynamics in favor of a balanced immune response. In **Figure 9** the effectiveness of a molecule that inhibited IKK activity (IKK-inhibitor) was simulated. From a biological standpoint, these molecules diffuse into the cytoplasm and bind to IKK triggering its deactivation. This process directly competed with the activation of the NF-kB complex through IKK and therefore attenuation in the pro-inflammatory response was observed. As such, despite the implications of high LPS concentration, the dynamics were reversed towards homeostasis. Qualitatively, this result agreed with experimental data that documented the potential of IKK inhibitors in treating inflammatory disorders [51].

Our model exhibited a bistable behavior which implied the co-existence of two steady states. Physiologically, such dynamics would reflect either a successful inflammatory resolution or the progression of a systemic inflammatory response syndrome. Such bistability is an essential characteristic of the non-linear dynamics of inflammation as suggested from various animal studies [36]. In an attempt to simulate the bistable behavior, a “switch” in the agent rules was employed. A switch was defined as a conditional procedure under which the output could diverge into different states based on a current set of inputs. In our simulation the switch was the production rate of TNF-a with regard to the energetic state of the macrophage. As the current energy value become lower than a certain threshold, the production of TNF-a via the transcription factor NF-kB was amplified, activating the switch. The rationale behind this rule was predicated upon the hypothesis that the activation of NF-kB, followed by the production of (P) response ultimately decrease the expression of genes that are involved in bio-energetic cellular processes [52]. On the other hand the number of anti-inflammatory (A) molecules raised the energy level and drove the cells to the “healthy” state, deactivating the switch. Many switch-like phenomena have been observed in biological systems [53].

In conclusion, an agent based modeling framework is proposed as an alternative modeling approach to study the complex, non-linear dynamics of acute human inflammation. We specifically proposed an agent based model that couples critical aspects of the host response to endotoxin. Predicated upon our prior research effort where a deterministic approach has been taken to couple extracellular signals and intricate signaling cascades with the transcriptional response level, the work discussed in this study explored the potential of an agent based modeling approach to improve our understanding of how a system gives rise to a response through its interacting molecules (or cells). Agent based models offer a promising approach in that they can express the dynamics through intuitive multiple interactions between the agents over time. A well known feature of ABMs is their ability to generate surprisingly complex and emergent behavior from very simple rules. However, this modeling framework is not without its own limitations. A key limitation of agent-based modeling has to do with the difficulty in applying a formal analysis to the relationship between the agent rules and the behavior of the system [8], [54]. Thus, in contrast to equation-based modeling for which analytical tasks (e.g. parameter sensitivity analysis, bifurcation analysis, e.t.c.) can be performed, the stochastic behavior of agents makes it extremely difficult to analyze how each parameter of an AMB simulation affects the output of ABM. To address this issue, researchers rely upon the principles of pattern-oriented analysis, in which patterns of dynamic behavior are used to relate the computational ABM to its real-world reference [55]. The downside of this characteristic, however, is that extensive computational power may be required to generate dense datasets amenable to statistical analysis. In this context, the high computational cost related to running ABM, as compared to equation-based models, may constrain the size of ABM implementations that can be run in the typical academic setting. However, researchers have started to explore “hybrid” model systems in order to increase the feasibility of large ABM-based simulations. Additionally, the recognition that both approaches (EBM and ABM) have their advantages and limitations has placed emphasis on cross-platform validation where some processes are simulated discretely while other processes are handled in a continuous simulation [56]. It is important to realize that such multi-modal approaches are complementary and ideally both would be used to provide a mathematical characterization of a complex dynamical system.

## Materials and Methods

### Human Endotoxin Model and Data Collection

The data used in this study were generated as part of the Inflammation and Host Response to Injury Large Scale Collaborative Project funded by the USPHS, U54 GM621119 [57], [58]. Human subjects were injected intravenously with endotoxin (CC-RE, lot 2) at a dose of 2-ng/kg body weight (endotoxin treated subjects) or 0.9% sodium chloride (placebo treated subjects). Following lysis of erythrocytes and isolation of total RNA from leukocyte pellets, [57], biotin-labeled cRNA was hybridized to the Hu133A and Hu133B arrays containing a total of 44,924 probes for measuring the expression level of genes that can be either activated or repressed in response to endotoxin. A set of 5,093 probe sets were characterized by significant variation (corresponding to 0.1% false discovery rate) across the time course of the experiment using the SAM software [59]. The data are publicly available through the GEO Omnibus Database (http://www.ncbi.nlm.nih.gov/geo/) under the accession number GSE3284. The data have been appropriately de-identified, and appropriate IRB approval and informed, written consent were obtained by the glue grant investigators [57].

In order to integrate high-throughput transcriptional data we recently introduced a systems level approach [32], [60] that decomposes high-dimensional microarray data into a critical set of dynamic features that are considered to be the elementary inflammatory responses triggered by the endotoxin stimulus in peripheral blood leukocytes (PBLs). Our fundamental assumption is that the transcriptional signatures capture the cellular dynamics in response to the inflammatory agent. These constitutive dynamics features are considered to be the “blueprints” of the orchestrated dynamics of the perturbed biological system and include the pro-inflammatory response; a later transcriptional event indicative of anti-inflammation and ultimately the energetic response. The potential of a physicochemical host response model that integrates transcriptional profiling, intricate signaling cascades and indirect response models is demonstrated in [33], [34]. Predicated upon the essential interactions that define the propagation of LPS signaling across the system, we opt to translate them into an integrated ABM framework.

### Developing an Agent Based Model of Endotoxin Induced Human Inflammation

The inflammatory response is activated when endotoxin is recognized by pathogen recognition receptors [61]. Such recognition process involves the induction of a signal transduction cascade that triggers downstream critical signaling modules for the activation of transcriptional factors that play a critical role for the transcriptional initiation of inflammatory genes. LPS molecules collide with their receptor, TLR4, on the surface of the macrophages. If the receptor is unoccupied, the LPS molecule will have a probability to bind to its signaling receptor, forming a complex. A receptor that is already bound to a LPS molecule will be unable to receive another one. The bound receptor is also considered activated, in that it will up-regulate the production of TNF-a molecules stimulating downstream intricate signaling cascades. Such a cascade involves the activation of kinase (IKK) activity, which in turn phosphorylates the inhibitor protein IkBa and leads to the activation of the transcription factor NF-kB. The transcriptional end result of this signaling pathway is the production of pro-inflammatory cytokines including IL-12, TNF-a, and IkBa. The IkBa molecules effectively terminate the pathway by forming an inactive complex with nuclear NF-kB in the cytosol. The production of IL-12 initiates the production of IL-4 molecules. These two cytokines populate the system and bind to their respective receptors on the macrophages or type T0 helper cells. The fate of Th-0 cells is determined by the number of either IL-4 or IL-12 on its surface receptors [28].

The pro- and anti-inflammatory mediator profiles (P and A) and the energetic response of the macrophages (E) were used as a primary indication of a constrained or unresolved inflammatory response. During the progression of systemic inflammation, pro-inflammatory (P) molecules specifically reflect the presence of IL-12 mediators that are circulating in the system. The primary reason for such a selection stems from the fact that the role of IL-12 has been implicated in the differentiation of Th-0 cells. However, each essential transcriptional signature (P, A, E) as it previously mentioned, serves as the aggregate signal that describes complex inflammatory reactions. Thus, (P) would qualitatively reflect the secretion of cytokines and chemokines such as TNFSF2 (TNF), IL1A, IL1B, CXCL1, CXCL2, CCL2, CXCL8 (IL-8) and CXCL10. Similarly, the anti-inflammatory arm of the system (A) reflects either the number of IL-4 molecules upon endotoxin simulation or mediators such as IL1RAP, IL1R2, IL10 and TNFRSF1A. We would like to comment that while these two quantities specifically measures the amount of a particular species, they are however a qualitative description to indicate the state of the system. By the same token, although the energetic response (E) is given as a quantity in “molecules” in the model, it is only a descriptive quality as a marker to track the state of the system and does not have a physical manifestation in physiology. The energetic response (E) refers to those transcriptional signatures that participate in the cellular bio-energetic processes, mainly in the ATP producing pathways [62] and is affected by the transcriptional activities of NF-kB, coupled with the anti-inflammatory cytokine response. Moreover, activated NF-kB, IKK, and IkBa molecules are the summation of activated population of respective species in all macrophages and LPS refers to the total amount of LPS in circulation, both bound and free. Regarding TNF-a, it refers to free TNF-a molecules.

### Agent Rules and Behaviors

Agents are the main components that follow specific instructions on how they should behave and interact with other agents. The types of agents are listed in **Table 2**. Each agent has its own properties that define the type of behavior and interactions that the agent is involved with. Different types of agents are grouped into different classes, e.g., a type of interleukin or stimulus. Some properties are present in many classes, e.g., degradation counter that determines when an agent disappears or die; or location reporter that informs the molecule its position with regard to another molecule. Other properties only pertain to a certain class, e.g., receptor sensitivity that dictates whether binding occurs, or macrophage energetic level that serves as a survival indicator. The “world” is defined by a coordinate system with boundaries that wrap around horizontally and vertically. Macrophages are placed randomly in the world, provided that there is no overlapping between each macrophage. The simulation is computationally intensive in that each macrophage cell alone is composed of more than 400 agents. For the purpose of reducing the computation time for each simulation, a 161 by 161 world and 4 macrophages were used for the experiments. After experimenting with a range of world sizes, we decided that the selected size was appropriate to accurately generate the dynamic profiles while allowing repeated simulations to run at a desirable pace.

**Table 2:** List of agents.

Cell types	Macrophage, Th-0, Th-1, Th-2
Stimulus, mediator	LPS, IL-12, IL-4, TNF-a
Receptors	TLR4, IL-12R, IL-4R, TNFR
Intracellular signaling molecules	IKK, NF-kB, IkBa
Cellular component	Plasma membrane, nucleus

As the model is executed, it performs a list of procedures in an order. The execution is an iterative process where each iteration represents a “tick” or a discrete time point. Each procedure governs the behavior of a specific class of agent; it contains instructions on how an agent should move, whether to bind to a receptor or “bounce” off of the cell membrane, etc. The instructions are conditional (rule) based (if-then) and may involve multiple agents, such as when two molecules bind together, both molecules' parameters change due to the binding, for instance, they now move in the same pattern. Moreover, the instructions are derived from literature regarding relevant mechanisms for LPS activation [35], [43], [63], intricate signaling cascades [64], [65], [66], [67], [68], [69], cytokine network [40], [41], [70] and cell (Th) differentiation [28], [31]. The specific rules that determine the behavior of the relevant agents are presented in **Table 1**.

The movement of LPS molecules is characterized by a random walk routine, namely, each molecule heads to a random direction and moves several steps forward. When one LPS molecule comes in contact with its signaling receptor on the surface of the macrophage, it will have a chance to bind to it. Once bound a molecule will no longer be moving freely; it will move in accordance with its counterpart. Such process triggers the production of the proximal inflammation-specific mediator, TNF-a causing a decrease in receptor's sensitivity. Receptor sensitivity determines the probability by which one LPS molecule will bind to its endotoxin receptor. Bound LPS molecules are degraded shortly, freeing up the receptor. Molecules of TNF-a also move randomly where they can diffuse into the cell (move past the cell membrane agents) and bind to their appropriate receptors either from the cytoplasm or from outside of the membrane. The activated receptor will trigger downstream a signal transduction cascade that stimulates IKK activity. Activated IKKs move randomly inside the cytosol while they are not capable in moving past the cell membrane agents or enter the nucleus region. They activate the NF-kB complex by dissociating the bound between NF-kB and its inhibitor, IkBa. This is achieved through the phosphorylation of the inhibitory protein IkBa where dissociated IkBa is therefore ubiquitinated and degraded by the proteasome. Activated NF-kB then moves into the nucleus region initiating the transcriptional machinery program which up-regulates the transcription of IkBa, of pro-inflammatory cytokines (P) followed by a decrease in the energetic state of the macrophage. Activated IkBas are capable of moving into the nucleus, binding to activated NF-kB molecules, and deactivate them, as they retrieve nuclear concentrations of NF-kB by forming an inactive complex in the cytoplasmic region.

The pro-inflammatory (P) agents are limited by cell membranes and their presence excites the production of anti-inflammatory cytokines (A) and the migration of undifferentiated T-helper cells (Th0) which are not present under conditions of no infec7tious challenge. However, the secretion of pro-inflammatory (P) molecules by macrophages during the progression of the inflammatory reaction induces Th0 cells to enter the “virtual world”. This event signifies the migration of cytokines into the spleen, within which the differentiation of Th0 cells takes place. In this model the role of T-helper cells is to regulate the feedback loops associated with pro-(P) and anti-inflammatory (A) mediators. Further, the binding of (A) molecules with their signaling receptor will stimulate an energy expenditure causing nearby bound TNF-a to degrade faster. In addition to Th0 cell type under conditions of an abundance of bound anti-inflammatory (A) cytokines with their receptors it will morph into a Th2 cell. If it happens that the presence of (P) molecules on the surface receptors to outweigh the (A) response, then Th0 will become of Th1 type which potentiates the pro-inflammatory response. Conversely, Th2 cell type response potentiates the secretion of anti-inflammatory cytokines (A).

In order to facilitate the translation from literature evidence into a programming language, it is necessary to provide a feasible framework that integrates disparate research into a conceptually valid scheme, taken into account the abstractness and limitations of the model. Some of the goals are outlined in literature [15] but in this model, we place emphasis on the potential mechanisms that drive complex responses identifying essential elements of the probed response.

### Model Calibration and Validation

Due to the inherent stochasticity of the ABM development, calibration is oftentimes performed on a trial and error basis. This process involves generating multiple sets of results by systematically varying the model parameters at each set. Also known as “parameter sweeping”, this process allows us to explore the possible behaviors of the model and determine which parameters will engender the patterns that best represent the behavior of interest. For instance, we first examine the signaling agents that will have the most leverage on repressing the inflammatory response. After each simulation, we adjust the parameters such as the production rate of P and TNF-a, the movement speed of LPS, or the probability that an interaction will occur between two colliding signaling molecules. These parameter values were manipulated so that the simulations lie in qualitative agreement with the self-limited inflammatory response. This implies that from among the multiple runs we select those that can effectively reproduce dynamic profiles associated with the successful elimination of the inflammatory stimulus within the first 2 hr post-endotoxin administration while followed by a subsequent transcriptional resolution within 24 hr. We define the parameters that can produce the self-limited profile as a basis set and based on this set we simulate the LPS dosage dependent responses. A set of parameters is considered satisfactory if the model is capable of simulating the dynamics of a self-limited inflammatory response (resolution within 24 hr post-LPS administration) as well as successfully generating the series of unconstrained (non-linear) responses as previously discussed in this paper. The results of the simulations are compared on a qualitative manner with our prior equation-based host response models as shown in Figure 4 and Figure 5 (dashed lines). While comparing the output of ABM with the output of the ODE, it should be noted that both modeling approaches are not characterized by the same network topology. Specifically, in the proposed ABM additional inflammatory mediators (molecules, cells) are considered when compared to the ODE model which may account for the observed variations in the simulated responses of the two modeling frameworks. However, albeit different in network topology, the two modeling frameworks predict responses (e.g. P, A, E) that lie in a good qualitative agreement.

This ABM is developed using NetLogo (Center for Connected Learning and Computer-Based Modeling, Northwestern University, Evanston, IL), a freeware that constructs agent based models.
